# Ramulus mori (Sangzhi) alkaloids improve intestinal oxidative damage and inflammation in DHEA-induced polycystic ovary syndrome rats via gut microbiota and metabolite modulation

**DOI:** 10.3389/fphar.2025.1701694

**Published:** 2026-01-22

**Authors:** Yanping Wang, Xianmei Jiang, Shuyi Wu, Qiaohui Wang, Dan Zuo, Biao Huang, Li Jian, Yu Yang, Yong Cai, Xingjian Wen, Ling Yao, Shan Geng

**Affiliations:** 1 Central Laboratory of the People’s Hospital of Dazu, The Affiliated Dazu’s Hospital of Chongqing Medical University, Chongqing, China; 2 Basic Medical College, Chongqing University of Chinese Medicine, Chongqing, China; 3 Clinical Nutrition Department, The Affiliated Dazu’s Hospital of Chongqing Medical University, Chongqing, China; 4 Department of Critical Care Medicine, The Affiliated Dazu’s Hospital of Chongqing Medical University, Chongqing, China; 5 Department of Endocrinology, The Affiliated Dazu’s Hospital of Chongqing Medical University, Chongqing, China; 6 Chongqing Academy of Chinese Materia Medica, Chongqing, China

**Keywords:** Ramulus mori (Sangzhi) alkaloids, PCOS, intestinal oxidative damage, inflammatorystatus, intestinal microbiome and metabolites, fenoldopam, bile acid metabolism

## Abstract

**Introduction:**

Intestinal dysbiosis, characterized by reduced diversity and enrichment of pro-inflammatory taxa, is implicated in the pathogenesis of polycystic ovary syndrome (PCOS). Ramulus mori (Sangzhi) alkaloids (SZ-A), approved in China for type 2 diabetes with broad metabolic effects, remain untested as a microbiota-targeted intervention for PCOS.

**Methods:**

In a dehydroepiandrosterone (DHEA)-induced rat model of PCOS, we evaluated the therapeutic efficacy of SZ-A and its underlying microbiota–metabolite interactions through integrated assessments of reproductive and endocrine–metabolic function, oxidative stress, inflammatory cytokines, and gut microbiota and serum metabolite profiles.

**Results:**

Relative to SD rats, PCOS rats showed approximately 10-fold higher cystic follicle burden and a one-third reduction in corpora lutea, with serum testosterone rising from 0.12 ± 0.08 to 0.27 ± 0.08 ng/mL, total bile acids falling from 34.22 ± 5.52 to 20.63 ± 4.94 μM, and HOMA-IR significantly increased (all p < 0.05). SZ-A treatment reduced cystic follicles, restored estrous cyclicity and luteal formation, and shifted testosterone, total bile acids, and HOMA-IR toward SD levels. At the molecular level, SZ-A appears to act by remodeling gut microbiota composition and serum metabolite profiles. SZ-A significantly shifted microbial β-diversity in PCOS rats while retaining a community dominated by Bacteroidetes and Firmicutes with *Lactobacillus* and *Treponema*_2 as key genera. Untargeted metabolomics identified 13 PCOS-associated serum metabolites that were significantly reduced after SZ-A treatment (p < 0.05), highlighting fenoldopam as a putative mediator of its beneficial effects on ovarian function and metabolic homeostasis. With respect to oxidative injury, serum malondialdehyde (MDA) levels in PCOS rats were approximately twice those of the SD group, while total antioxidant capacity (T-AOC) and the activities of superoxide dismutase (SOD) and glutathione peroxidase (GSH-Px) were significantly reduced (p < 0.05); treatment with SZ-A markedly attenuated these alterations (p < 0.05). Besides, it suppressed systemic inflammation by reducing interleukin-6 (IL-6), interleukin-1β (IL-1β) and tumor necrosis factor-α (TNF-α) levels in serum and relevant tissues (p < 0.05).

**Discussion:**

Collectively, these findings indicate that SZ-A alleviates PCOS by attenuating intestinal oxidative stress and normalizing gut microbiota–metabolite interactions, and highlight fenoldopam as a potential effector, supporting SZ-A as a promising therapeutic candidate for PCOS.

## 1 Introduction

Polycystic ovarian syndrome (PCOS) is a multifaceted ailment that is related to an endocrine reproductive disorder, which arises from various factors in women (Helvaci and Yildiz, 2025). PCOS primarily affects females in the reproductive age (Stener-Victorin et al., 2024). PCOS is primarily characterized by an excess of androgens, ovarian insufficiency, and the development of polycystic ovaries (Teede et al., 2023). The quality of life of individuals suffering from PCOS is often significantly poorer than that of their healthy counterparts (Wilson et al., 2020; Ligocka et al., 2024). Despite the fact that genetic, neuroendocrine, and oxidative stress have been identified as contributing factors to PCOS, the exact mechanism of this condition is still unknown.

Previous research indicates that the intestinal microbial balance has a crucial contribution to the pathogenesis of PCOS (Hanna et al., 2025). Previous reports have confirmed that imbalances in the intestinal microbiome might be related to the various manifestations of PCOS. Various factors, including IR, serum levels of hormones, and obesity, are proposed to influence the diversity and composition of the intestinal microbes in individuals suffering from PCOS (Mei et al., 2025). Other reports confirm that the intestinal microbiota of overweight teenagers with PCOS vary from those of overweight teenagers without PCOS, and these variations are related to the concentration of testosterone and indicators of metabolic imbalances (Jobira et al., 2020). Altogether, these observations emphasize the significance of maintaining a healthy intestinal microbiome for the therapeutic management of PCOS.

Intestinal dysbiosis reshapes key metabolite signals, including short chain fatty acids (SCFAs), bile acids, and tryptophan derivatives, which influence folliculogenesis, steroidogenesis, and immune and inflammatory homeostasis, thereby modulating the reproductive and metabolic phenotype of PCOS (Zhao et al., 2025; Li C. et al., 2025; Senthilkumar and Arumugam, 2025). Microbiota derived SCFAs promote follicular maturation and ovulation while limiting granulosa cell apoptosis (Xu et al., 2025); notably, butyrate alleviates granulosa cell inflammation and corrects epigenetic and transcriptional dysregulation in rodent and cellular models (Liu K. et al., 2023). At the same time, disturbances in the gut bile acid pool and in FXR and TGR5 signaling are associated with glucose and lipid dysmetabolism, impaired incretin secretion, and systemic inflammation, suggesting that restoration of microbial bile acid transformation and receptor pathways may yield dual metabolic and reproductive benefits (Młynarska et al., 2024; Li J. et al., 2025). Along the tryptophan pathway, indole 3 propionic acid (Mehta et al., 2025) improves DHEA induced PCOS phenotypes through the AhR-NLRP3 axis (Li Z. et al., 2025), whereas elevated kynurenine and activation of the AhR-PCSK9 pathway relate to lipid and inflammatory imbalance, together nominating the microbe to tryptophan to AhR signaling module as an actionable target (Wang et al., 2025). Despite growing evidence linking the gut microbiome to PCOS, the relevant metabolic signaling networks, causal mechanisms, and microbial biomarkers suitable for translation remain incompletely defined. Deeper mechanistic dissection of the gut ovary axis will inform stratified interventions and the development of innovative therapies for PCOS.

Ramulus Mori (Sangzhi) Alkaloids (SZ-A) is an ancient Chinese medicine that comprises an array of active polyhydroxy alkaloids that can be isolated from the branches of mulberry plants (Wu et al., 2025). SZ-A has been approved for the management of type 2 diabetes mellitus (T2DM) by the National Medical Products Administration (NMPA) of China (Approval No.: Z20200002). SZ-A constitutes greater than half of the extract prepared from mulberry twigs, and predominantly comprises fagomine (FA), 1-deoxynojirimycin (DNJ), and 1,4-dideoxy-1,4-imino-D-arabinitol (DAB) (Liu et al., 2021; Qu et al., 2021; Liu Z. et al., 2023; Zhao et al., 2024). Previous studies have demonstrated that intestinal microorganisms and their secreted metabolites play significant roles in the development of nonalcoholic fatty liver disease (NAFLD), obesity, and T2DM (Liu et al., 2021; Chen et al., 2022; Sun et al., 2022; Schnabl et al., 2025). However, it remains to be determined whether SZ-A can alleviate PCOS by altering the composition of the intestinal microbiome and the metabolic profiles.

To this end, we constructed a dehydroepiandrosterone (DHEA)-stimulated rat model of PCOS, and the consequences of treatment with SZ-A on the ovarian activity, levels of oxidative stress, IR, and constitution of the intestinal microbes were investigated. We employed the 16s amplicon sequencing method and non-targeted metabolomics analyses to determine the changes in the intestinal microbes and the metabolic profiles of the rat model of PCOS. The findings obtained herein provide novel insights for the alleviation of PCOS by treatment with SZ-A.

## 2 Materials and methods

### 2.1 Chemical reagents and primary antibodies

The primary antibodies against pro-caspase-12, caspase-12, caspase-3, cleaved caspase-3, and glyceraldehyde-3-phosphate dehydrogenase (GAPDH) were purchased from Abcam (catalog numbers: #ab8117, #ab62484, #ab32351, #ab32042, and #ab181602, respectively; Abcam Inc., USA). Powdered SZ-A (lot number: J202012017) was generously supplied by Beijing Wehand-Bio Pharmaceutical Co., Ltd. (Beijing, China). The powder contained polyhydroxy alkaloids (57.0%), comprising 36.95% DNJ, 7.79% DAB, and 7.09% FA. Fenoldopam hydrochloride was procured from Aladdin Biochemical Technology Co., Ltd (catalog number: #67227-56-9). DHEA was procured from Aladdin Biochemical Technology Co., Ltd (catalog number: #53-43-0, Shanghai, China). The kit for determining the total antioxidant capacity (T-AOC) was procured from Beyotime Institute of Biotechnology Co., Ltd. (catalog number: #C1091, Shanghai, China). The kits used for measuring the activity levels of the enzymes superoxide dismutase (SOD), glutathione peroxidase (GSH-PX), malonyldialdehyde (Alhajeri et al., 2022) were procured from Nanjing Jiancheng Bioengineering Institute (catalog numbers: #A001-3, #A006-2-1, and #A003-1, respectively; Nanjing, China). The enzyme-linked immunosorbent assay (ELISA) kits for interleukin-6 (IL-6), IL-1β, and tumor necrosis factor alpha (TNF-α) were procured from Abcam (catalog numbers: #ab234570, #ab100768, and #ab236712, respectively; Abcam Inc., USA). The kits for TUNEL and DAB staining were procured from Wuhan Boster Biological Technology Co., Ltd. (catalog numbers: #MK1015 and #AR1002, respectively; Wuhan, China). Wright’s stain kit, RIPA lysate (Medium), and Hoechst33342 were procured from Beyotime Institute of Biotechnology Co., Ltd. (catalog numbers: #C0135, #P0013C, and #C1022, respectively; Shanghai, China).

### 2.2 Experimental animals

Animal research obtained approval from Chongqing Medical University’s Ethics Committee. 3-week-old female Sprague-Dawley (SD, 40-60 g) rats (Zhang W. et al., 2024; Mallya et al., 2025) were acquired from the Animal Center at our institute, and were housed under controlled conditions of temperature of 23 ° ± 2 °C with relative humidity of 65% and 12 h dark light cycle, with *ad libitum* access to standard chow and water. Subcutaneous injections of DHEA (60 mg/kg) dissolved in sesame oil (0.2 mL) were administered to 3-week-old female SD rats on a daily basis for inducing PCOS (Kim et al., 2018; Shi et al., 2019), while the rats in the control setup only received injections of sesame oil (0.2 mL) on a daily basis (SD group). After 21 days, 5 mL saline (PCOS group) or 200 mg/kg SZ-A dissolved in 5 mL saline (PCOS+SZ-A group) was administered via oral gavage for an additional 12 days (Liu et al., 2021; Chen et al., 2022; Liu Z. et al., 2023). Some of the rats were subsequently euthanized and the serum samples, ovaries, and intestines were collected. Anesthesia was administered through right intraperitoneal injection using Delivector™ Avertin (DW3101, Dowobio, Shanghai, China) (Underwood and Anthony, 2020). Fresh samples of feces were simultaneously obtained from the colonic tissues during this time. The samples were transferred to sterile 5 mL EP tubes that were subjected to rapid snap-freezing with liquid nitrogen and stored at a temperature of −80 °C. The remaining animals were used for studying the mean estrous cycle over the following 7–10 days.

Animals were housed and the PCOS rat model was established as described above, while the rats in the control setup only received injections of sesame oil (0.2 mL) on a daily basis (SD group). After 21 days, 5 mL saline (PCOS group) or 100 mg/kg Fenoldopam dissolved in 5 mL saline (PCOS+Fenoldopam group) was administered via oral gavage for an additional 12 days (Hanton et al., 1995). Some of the rats were subsequently euthanized and the serum samples, ovaries, and intestines were collected. Euthanasia and specimen preservation were performed as previously described.

### 2.3 Determination of estrous cycle

Vaginal smears were obtained on a daily basis from days 1–10 following the second treatment regimen. Then the estrous stage was ascertained by examination of the primary cell types in the collected vaginal smears with Wright’s staining kit, under a microscope (Yang et al., 2024).

### 2.4 Determination of the serum levels of biochemical markers

The serum levels of testosterone and total bile acids were determined using the respective assay kits (catalog numbers #H090-1-2 and #E003-2-1, respectively; Nanjing Jiancheng Bioengineering Institute, Nanjing, China). Additionally, the levels of blood glucose were assessed with ELISA kits purchased from Comin Biotechnology Co., Ltd (catalog number: #XT-1-Y, Suzhou, China). And serum insulin kit were provided by Biospes Co., Ltd (#BEK1243, Chongqing, China). The homeostatic model assessment of IR (HOMA-IR) index was calculated using the following formula: [levels of fasting serum insulin (lU/mL)] * [levels of fasting glucose (mmol/L)]/22.5. The oral glucose tolerance tests (OGTTs) and insulin tolerance tests (ITTs) were conducted in accordance with the protocol described in a previous study (Geng et al., 2021). Each of the experimental groups comprised six rats.

### 2.5 Tissue preparation for histological analyses

Ovarian tissue was collected from rats, washed and stored in formalin before immersion in paraffin. The ovaries were cut into 5 micron thick sections and incubated with hematoxylin and eosin (H&E) or TUNEL stain. Each experimental group contained at least 3 rats.

### 2.6 Apoptosis analysis-tunel stain

Initially, tissue sections were subjected to a dewaxing step that included soaking in xylene three times for 5 min each time, followed by rehydration in 100%, 90% and 70% ethanol for 5 min, followed by rinsing with distilled water for 5 min. Subsequently, sections were treated with Proteinase K (no DNase, 20 ug/mL) and incubated at 37 °C for 15 min. Sections were washed five times with phosphate buffered saline (PBS), after which TUNEL reaction mixture was added to the sections and incubated at 37 °C in the dark for 90 min. Tissues were then washed three times with PBS, and finally stained by incubation with DAB or Hoechst33342 for 10 min, washed three more times with PBS, and observed immediately.

### 2.7 Western blotting

In the method outlined in a previous study (Geng et al., 2021), the ovaries were first rolled, the protein was extracted using RIPA lysate and placed on ice for 30 min, the tissue homogenate was then centrifuged at 4 °C with a centrifugal force of 12000×g for 20 min, and the obtained supernatant was finally subjected to protein immunoblotting analysis.

### 2.8 Untargeted metabolomics detection of plasma

Fasting blood samples were collected from rats in the experimental group and placed into 5-mL vacuum blood collection tubes. Chelated with EDTA. The collected blood samples were centrifuged at a temperature of 4 °C with a centrifugal force of 1500×g for 15 min, and then 150 μL aliquots were taken. Frozen at minus 80 °C, and the plasma samples were preserved for use in ultra-performance liquid chromatography-quadrupole time of flight mass spectrometry (UPLC-Q- TOF/MS) system for subsequent analysis, the frozen plasma was thawed at a temperature of 4 °C, and deproteinization was achieved by mixing the plasma sample (100 μL) with a solution of methanol and acetonitrile (volume ratio 1: 1,400 μL) at low temperatures. The deproteinized plasma sample was then centrifuged at a temperature of 4 °C with a centrifugal force of 14000×g for 15 min, and then the resulting supernatant was volatilized using a vacuum centrifuge. For sample reconstitution, the vaporized supernatant was mixed with a solution of acetonitrile and water (1:1, v/v; 100 μL). All the LC-MS data were analyzed by Shanghai Applied Protein Technology Co., Ltd (China). The details of the methodology have been documented in a previous study (Meng et al., 2023). To ensure analytical robustness and that the quality of untargeted metabolomics data met acceptable standards, pooled quality control (QC) samples were prepared by combining equal volumes of the study specimens. QC samples were injected at the beginning of each batch to condition the analytical platform and subsequently after every six study injections to monitor system performance. Metabolite features with a QC detection rate <80% and a relative standard deviation (RSD) > 30% were excluded from further analysis (Wang et al., 2022).

### 2.9 Gut microbiota analysis

The CTAB/SDS approach was used to extract sample DNA for analyzing the intestinal microbiota. The purity and the concentration of the DNA were analyzed using agarose gels of 1% concentration. Based on the concentration of the extracted DNA, it was diluted with appropriate amounts of sterile water to prepare a DNA solution of concentration 1 ng/μL. The primers for the V3–V4 (341F–806R) region of the 16S RNA gene, V9 (1380F–1510R) region of the 18S RNA gene, and the ITS1 gene (ITS1F–ITS2R) were used in this study. The genes encoding the 16S/18S rRNAs were amplified using specific primer pairs with barcodes. The total PCR volume was 30 μL, and comprised 15 μL of Phusion® High-Fidelity PCR Master Mix (New England Biolabs, USA), the forward and reverse primers (each 0.2 μM), and the template DNA (approximately 10 ng). The process of PCR amplification involved an initial denaturation step for 1 min at 98 °C, subsequent denaturation at 98 °C for 10 s (30 cycles), 30 s of annealing at 50 °C, 60 s of elongation at 72 °C, and a final extension for 5 min at 72 °C. Equal quantities of 1X loading buffer and SYB green dye were subsequently added to the products obtained by PCR amplification and electrophoresed on agarose gels of 2 concentration to visualize the results. Samples with distinct bands at 400–450 bp were selected for subsequent examination. The amplified DNA fragments obtained by PCR were subjected to purification using an AxyPrepDNA Gel Extraction Kit (Axygen Biosciences, USA). An NEB Next® Ultra™ DNA Library Prep Kit for Illumina (NEB, USA) was employed for preparing the sequencing libraries, according to the protocol provided by the manufacturer, along with the addition of index codes. A Qubit@ 2.0 Fluorometer (Thermo Scientific, USA) and an Agilent 2100 Bioanalyzer were employed for analyzing the quality of the sequencing libraries. A NovaSeq 6000 sequencing platform (Illumina Inc., USA) was used to sequence the prepared libraries, which generated 250 bp-long paired-end reads. All the data were analyzed by Shanghai Applied Protein Technology Co., Ltd. Clean sequence reads were imported to qiime2, and variant calling was cariied outusing DADA2, Sequences are clustered at 100% similarity and each de-duplicated sequence produced is called ASVs. Species annotation was performed using a pretrained Naive Bayes classifier, which was aligned with the SILVA 138 reference database. At the same, using QIIME2 software, samples were evaluated for Alpha Diversity Index: species abundance were estimated by Chao1 and Observed Species indexes, and microbial diversity were estimated by Shannon and Simpson indexes. Rarefaction curves were generated based on these metrics. Graphical representation of the relative abundance of microbial composition from phylum to species can be visualized using Krona chart. Venn, barplot and heatmap were used to showing microbial community composition among samples and groups. Beta diversity were visualized through PCA, PCoA and NMDS. To identify differences of microbial communities among different groups, ANOSIM and adonis were performed based on the Bray-Curtis dissimilarity distance matrices. To confirm differences in the abundances of individual taxonomy between the two groups, STAMP software was utilized. LefSe was used for the quantitative analysis of biomarkers within different groups.

### 2.10 Data and statistical analysis

All experiments subjected to statistical tests were conducted aminimum of three times as biological replicates. No data were excluded from the analyses. Statistical analyses were performed using Prism software version 10.1 (GraphPad). One-, or two-way ANOVA followed by Tukey’s test was used for comparisons among multiple groups. All the results are expressed as the means ± SEM. Statistical significance was set at p < 0.05. The correlation between the levels of metabolites in the fecal samples and the abundance of bacterial genera were analyzed by Spearman’s correlation analysis using the cor. test tool in the R statistical software. The microbial genera (P < 0.05) and the host metabolites (P < 0.05 and VIP >1) that exhibited significant differences across the groups were subjected to correlation analysis.

## 3 Results

### 3.1 SZ-A mitigates ovarian insufficiency and cellular apoptosis in the rat model of PCOS

**FIGURE 1:**
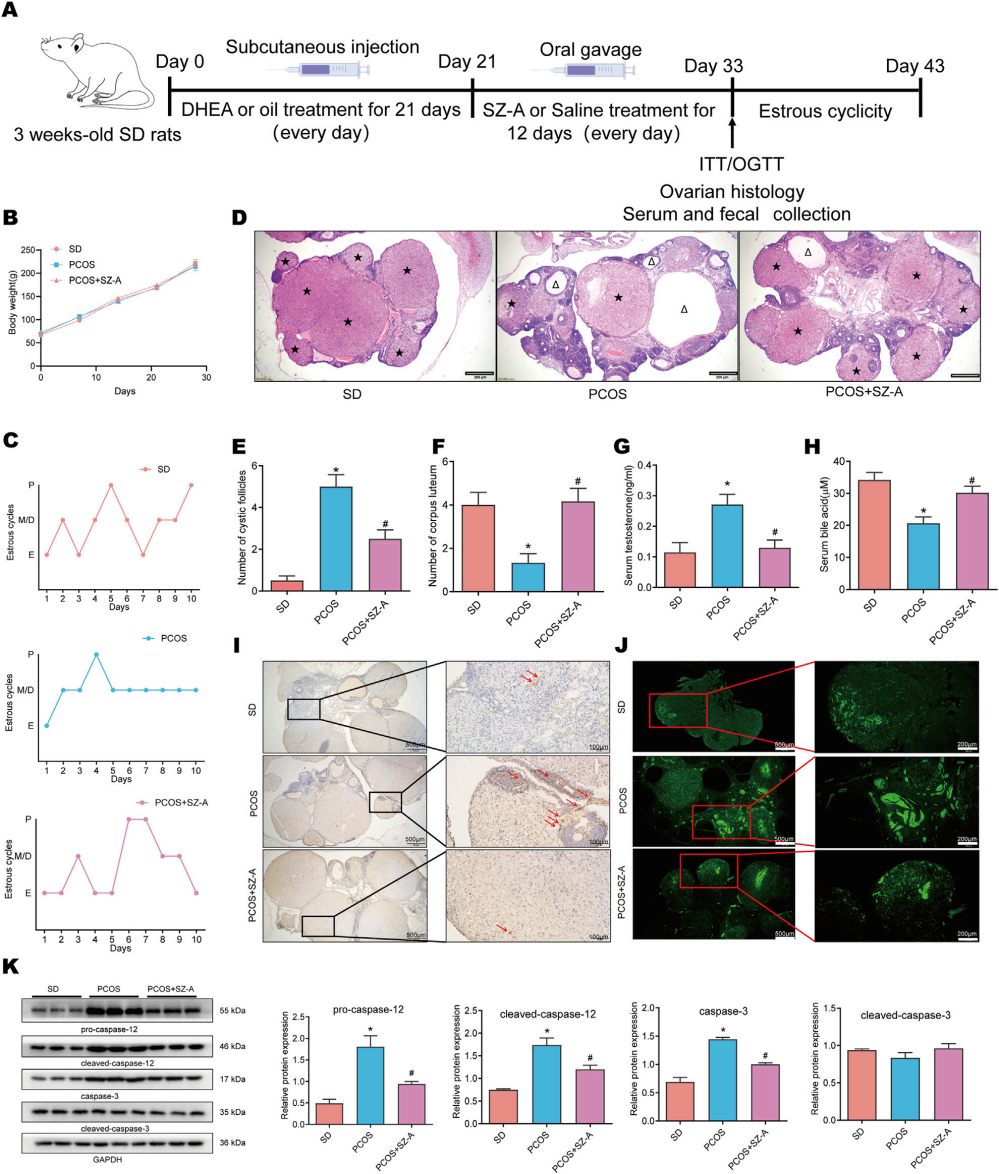
SZ-A alleviates ovarian dysfunction in dehydroepiandrosterone (DHEA)-induced PCOS rats. Female rats were randomly divided into three groups: SD, PCOS, PCOS +SZ-A. In this experiment, female SD rats were treated with DHEA or oil for 21 days, followed by intraperitoneal injection of SZ-A (200 mg/kg) or PBS, and then treated for 12 days. **(A)** Flow chart. **(B)** Body weight. **(C)** The estrous cycles were determined after another 10 days. **(D)** Representative ovarian sections stained with H&E (scale = 200) μ M; △ represents cystic follicles; ★ represents corpus luteum, and determine the number of cystic follicles **(E)** and corpus luteum **(F)** from these stained sections. Serum testosterone **(G)** and bile acid **(H)** levels were measured. **(I)** TUNEL staining of representative ovary sections. Red arrows point to TUNEL-positive cells. **(J)** Representative images from immunofluorescence TUNEL staining are presented. These sections have been stained for TUNEL (green). Scale bars are marked on the figures. **(K)** Western blot was used to detect the expression levels of Pro-caspase-12,Caspase-12,Caspase-3,Cleaved Caspase-3 in ovarian tissue lysates, and GAPDH was used as the loading control. Values are shown as the mean ± SEM (n = 6 rats).*represents significance compared to the SD group (*p < 0.05), # represents significance compared to the PCOS group (#p < 0.05). One-way ANOVA followed by Turkey’s test was used for **(E–H)** and **(K)**.

The protocol of the experiments is depicted in Figure 1A. The animals were randomly assigned to three experimental groups, namely, the SD, PCOS, and PCOS+SZ-A groups. As depicted in Figure 1B, the body weights were comparable across the different treatment groups on day 33, which was the end of the testing period. The influence of DHEA and/or SZ-A on ovarian function was determined by monitoring the estrous cycles. The estrous cycles of the rats in the SD group consistently lasted for 5–6 days (Figure 1C, upper panel), while the majority of the animals in the PCOS group remained in the diestrus phase (Figure 1C, middle panel). This irregularity in estrous cycles was alleviated following SZ-A treatment (Figure 1C, lower panel). H&E staining was subsequently performed to determine the variations among the ovarian pathologies of the different treatment groups. Analyses of ovarian cystic follicles and corpora lutea revealed that, compared with the SD group, rats in the PCOS model displayed a marked increase in cystic follicles and a reduction in corpora lutea (Figure 1D). Quantitatively, the number of cystic follicles in the PCOS group was about 10 times that of the SD group, whereas the number of corpora lutea was approximately one-third of that in the SD group (Figures 1E,F). In contrast, the administration of SZ-A reduced the cystic follicular count but elevated the count of corpora lutea (Figures 1D–F). The serum levels of testosterone were markedly higher in the PCOS group than in the SD group (0.27 ± 0.08 vs. 0.12 ± 0.08 ng/mL; mean ± SD). Administration of SZ-A significantly reduced serum testosterone in PCOS rats to 0.13 ± 0.06 ng/mL, approaching SD levels (Figure 1G). Evidence indicates that individuals with PCOS commonly have decreased levels of deoxycholic acid and taurine deoxycholic acid, impacting bile acid profile and metabolism (Yang et al., 2024). In SD rats, serum bile acids measured 34.22 ± 5.52 μM, while DHEA treatment (the PCOS group) markedly lowered levels to 20.63 ± 4.94 μM, whereas administration of SZ-A on the PCOS background resulted in a smaller decrease to 30.17 ± 4.92 μM (Figure 1H). The results of TUNEL staining revealed that treatment with SZ-A significantly downregulated the count of cells undergoing apoptosis in the rats with PCOS (Figures 1I,J). The ovarian expression levels of pro-caspase-12, cleaved caspase-12, and cleaved caspase-3 were higher for rats with PCOS, but this increase was attenuated following treatment with SZ-A (Figure 1K). Taken together, these observations imply that SZ-A mitigated ovarian insufficiency and mitigated the pathological injuries to the ovaries of rats with PCOS.

### 3.2 Moderate improvement in glucose tolerance in PCOS following treatment with SZ-A

**FIGURE 2:**
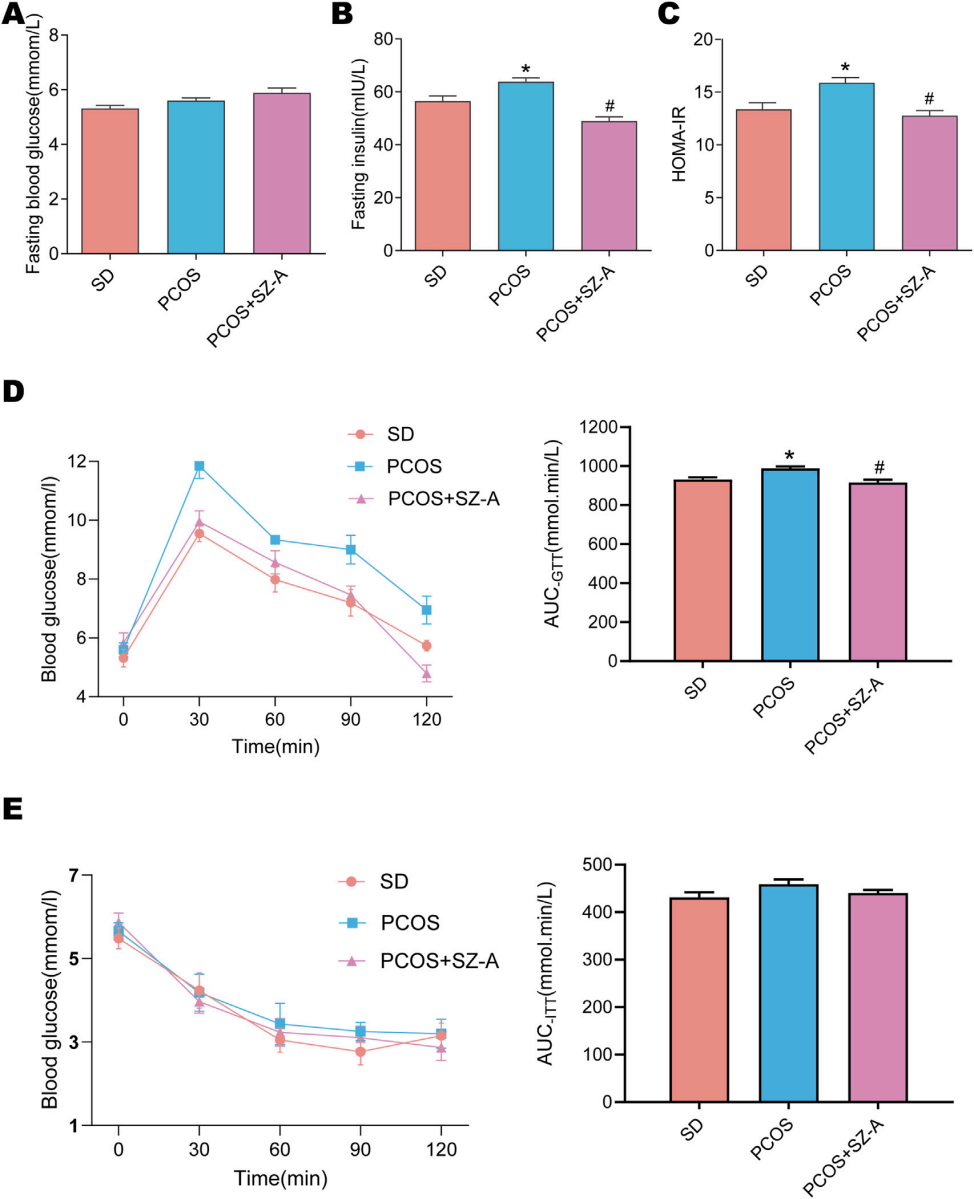
Effects of SZ-A on glucose tolerance and insulin sensitivity in PCOS rats. After 12 h of fasting, Blood glucose levels **(A)** and insulin **(B)** were measured in SD, PCOS, and PCOS+SZ-A groups of rats (n = 6). The steady-state model evaluation of insulin resistance (HOMA-IR) index **(C)**. Oral glucose tolerance tests (OGTTs) **(D)** and insulin tolerance tests (ITTs) **(E)** were performed on three groups of rats. Calculate the area under the curve (AUC) corresponding to blood glucose levels in each group. Values are shown as the mean ± SEM (n = 6 rats).*represents significance compared to the SD group (*p < 0.05), # represents significance compared to the PCOS group (#p < 0.05). One-way ANOVA followed by Turkey’s test was used for all results.

As PCOS is closely related to irregularities in the levels of serum metabolites, the insulin sensitivity of the rats in the different experimental groups and their glucose regulation were evaluated in this study. Despite the fact that the variations among the fasting blood glucose levels of the different treatment groups were not significant (Figure 2A), the levels of fasting serum insulin (Figure 2B) and HOMA-IR values (Figure 2C) in the PCOS group were higher than those of SD rats. Treatment with SZ-A significantly reduced the levels of fasting insulin and the HOMA-IR values of the group with PCOS (Figures 2B,C). The results of the OGTTs demonstrated a delay in glucose clearance and that the area under the curve was elevated for the PCOS group, indicating a decline in glucose excretion capacity. SZ-A administration ameliorated this abnormality in glucose tolerance (Figure 2D). Insulin Tolerance Tests (ITTs) illustrated that all three groups of rats responded equally well to insulin (Figure 2E).

### 3.3 SZ-A alleviates intestinal and systemic oxidative injury and inflammatory status of rats with PCOS

**FIGURE 3:**
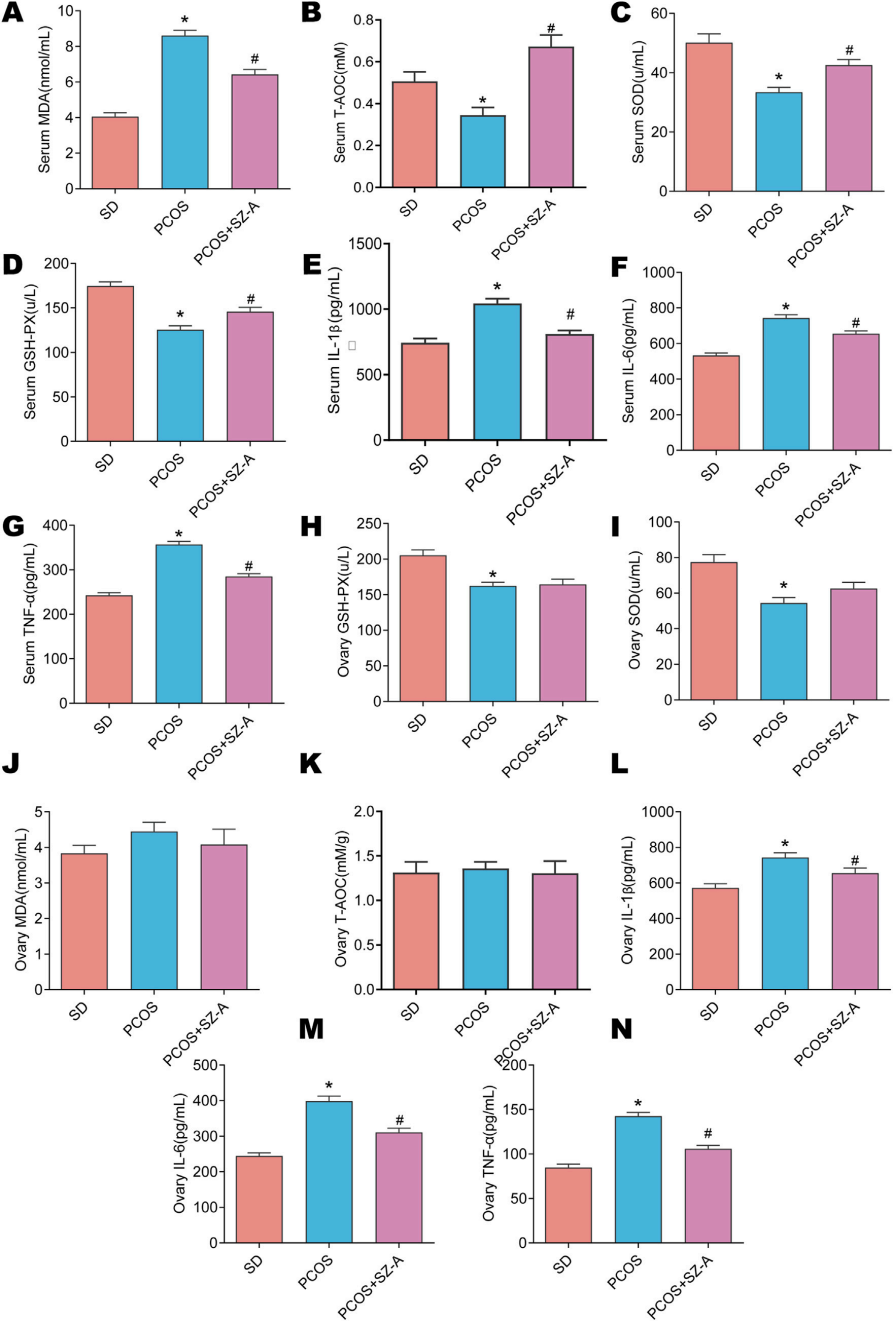
Effects of SZ-A on systemic and ovarian oxidative stress in PCOS rats. The serum levels of total antioxidant capacity T-AOC **(A)**, glutathione peroxidase (GSH-PX) **(B)**, malondialdehyde (Alhajeri et al., 2022) **(C)**, SOD activity **(D)**, interleukin-1β (IL-1β) **(E)**, Interleukin-6 (IL-6) **(F)** and tumor necrosis factor alpha (TNF-α) **(G)** were measured. The level of T-AOC **(H)**, GSH-PX **(I)**, MDA **(J)**, SOD activity **(K)**, IL-1 β **(L)**, IL-6 **(M)** and TNF-α **(N)** in ovarian lysates were determined. Values are shown as the mean ± SEM (n = 6 rats).*represents significance compared to the SD group (*p < 0.05), # represents significance compared to the PCOS group (#p < 0.05). One-way ANOVA followed by Turkey’s test was used for all results.

The serum levels of the biomarkers of oxidative were determined to assess the effect of SZ-A on systemic oxidative damage. Relative to SD controls, DHEA-induced PCOS rats exhibited a pronounced oxidative-stress phenotype, which serum MDA was approximately twofold higher (SD: 4.06 ± 0.55 nmol/mL), accompanied by reductions in serum T-AOC and the activities of GSH-Px and SOD. Treatment with SZ-A significantly reduced the serum levels of MDA but increased the serum T-AOC and the enzymatic activities of GSH-Px and SOD in rats with PCOS (Figures 3A–D). The serum concentrations of IL-1β, IL-6 and TNF-α in the PCOS rats were elevated compared to the serum levels in SD rats, while SZ-A significantly reduced the concentrations of these inflammatory factors in the PCOS group (Figures 3E–G). The ovarian levels of biomarkers of oxidative injury were further analyzed. In the ovaries of the PCOS group, the activities of GSH-Px and SOD were decreased relative to the SD group, whereas MDA levels and T-AOC did not differ significantly. SZ-A treatment did not alter ovarian T-AOC, SOD or GSH-Px activities, or MDA levels compared with the PCOS group (Figures 3H–K). Additionally, the ovarian concentrations of IL-1β, IL-6 and TNF-α in the PCOS group were higher than those of the SD group, while SZ-A significantly reduced the concentrations of pro-inflammatory factors in the ovaries of the PCOS group (Figures 3L–N). These findings suggest that SZ-A does not directly impact ovarian oxidative stress. As the intestinal microbiome has a crucial function in the emergence and advancement of PCOS, we additionally analyzed if SZ-A affects the redox state and inflammation in the intestinal tract of PCOS rats. SZ-A notably enhanced the serum T-AOC and enzymatic activities of GSH-Px and SOD in the rats with PCOS (Supplementary Figures S1A–C). Furthermore, the concentrations of MDA, IL-1β, IL-6 and TNF-α in the intestinal tissues were significantly upregulated in the animals with PCOS than those of the SD group, and the increased concentration of these factors was mitigated by SZ-A (Supplementary Figures S1D–G). These findings therefore revealed that treatment with SZ-A reduced oxidative stress in the intestinal tissues of rats with PCOS.

### 3.4 SZ-A modulates the dysbiosis of intestinal microbes in rats with PCOS

**FIGURE 4:**
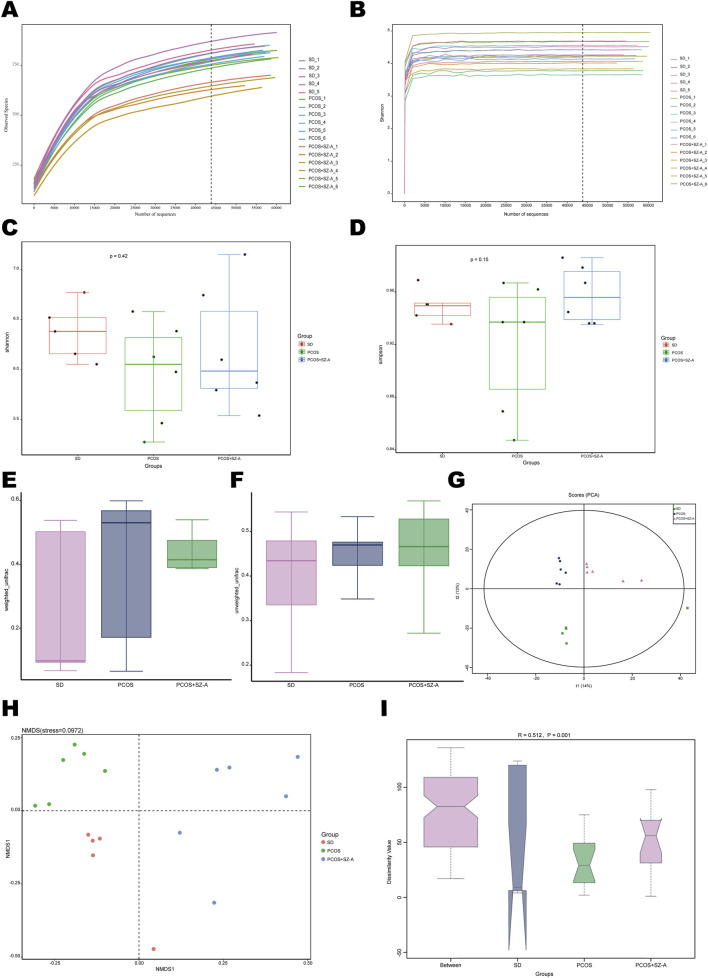
Effect of SZ-A on alpha diversity of gut microbiota. Analysis of gut microbial diversity was performed on the basis of 16S rDNA sequencing. Rarefaction curves **(A)** and Shannon index **(B)** are presented. Shannon index **(C)** and Simpson index **(D)** were used to describe alpha diversity of gut bacterial assemblages in the rats receiving different treatments. Bacterial community compositional similarity was evaluated by beta diversity. **(E)** Box plots based on weighted UniFrac beta diversity with P = 0.057 (t-test) and P = 0.043 (double wilcox). **(F)** Block diagram based on unweighted UniFrac beta diversity (P < 0.001). **(G)** PCoA analysis based on unweighted UniFrac distance and nonmetric multidimensional scaling (NMDS) plots based on Jaccard dissimilarity **(H)** are presented. Analysis of similarities (Anosim) was used to detect differences between the groups **(I)**. n = 5 rats in SD group, n = 6 rats in other groups, values are presented as means ± SD.

The 17 fecal samples collected in this study were subjected to analyses by 16S rDNA sequencing to assess the alterations in the abundance and composition of the intestinal microbes following the administration of SZ-A. Analysis of the sequencing data for the V3–V4 region of bacterial 16S rDNA revealed that the quality and volume of the sample were adequate. Analysis of species evenness and richness using the species accumulation and rank abundance curve indicated that the samples were suitable for further analyses. The diversities and abundances of the intestinal microbiome were subsequently assessed from the rarefaction curve and Shannon curve, respectively (Supplementary Figures S2A,B). The Shannon and Simpson alpha diversity indices were determined to measure the diversity of intestinal microbiota (Figures 4A,B). The rarefaction curves plateaued at 25,000 sequences, indicating sufficient sequencing data. Analysis of the Shannon curves similarly revealed that sequencing comprehensively accounted for the microbial diversity in the samples. Surprisingly, the alpha diversities did not exhibit significant variations between the groups treated with DHEA or SZ-A (Figures 4A–D). These findings implied that treatment with DHEA or SZ-A did not significantly alter the alpha diversities of the intestinal microbiomes of the animals in the PCOS group. The diagram box, constructed using weighted and unweighted UniFrac distances, indicated that there were significant variations among the β-diversities of the intestinal microbiomes of the three experimental groups (P < 0.05) (Figures 4E,F). The diversity of the intestinal microbiome across the different treatment groups was determined using the unweighted pair group strategy, unweighted principal coordinate analysis (PCoA), and the nonmetric multi-dimensional scaling (NMDS) method based on unweighted distance matrices. The results of NMDS and PCoA further revealed that there were variations in the overall composition of the intestinal microbiomes among the three rat cohorts (Figures 4G,H), and the Permanova/Anosim assessment confirmed the significance of these distinctions (Figure 4I). Taken together, these findings indicate that the gut microbiota’s β-diversity is influenced by the administration of DHEA and SZ-A.

**FIGURE 5:**
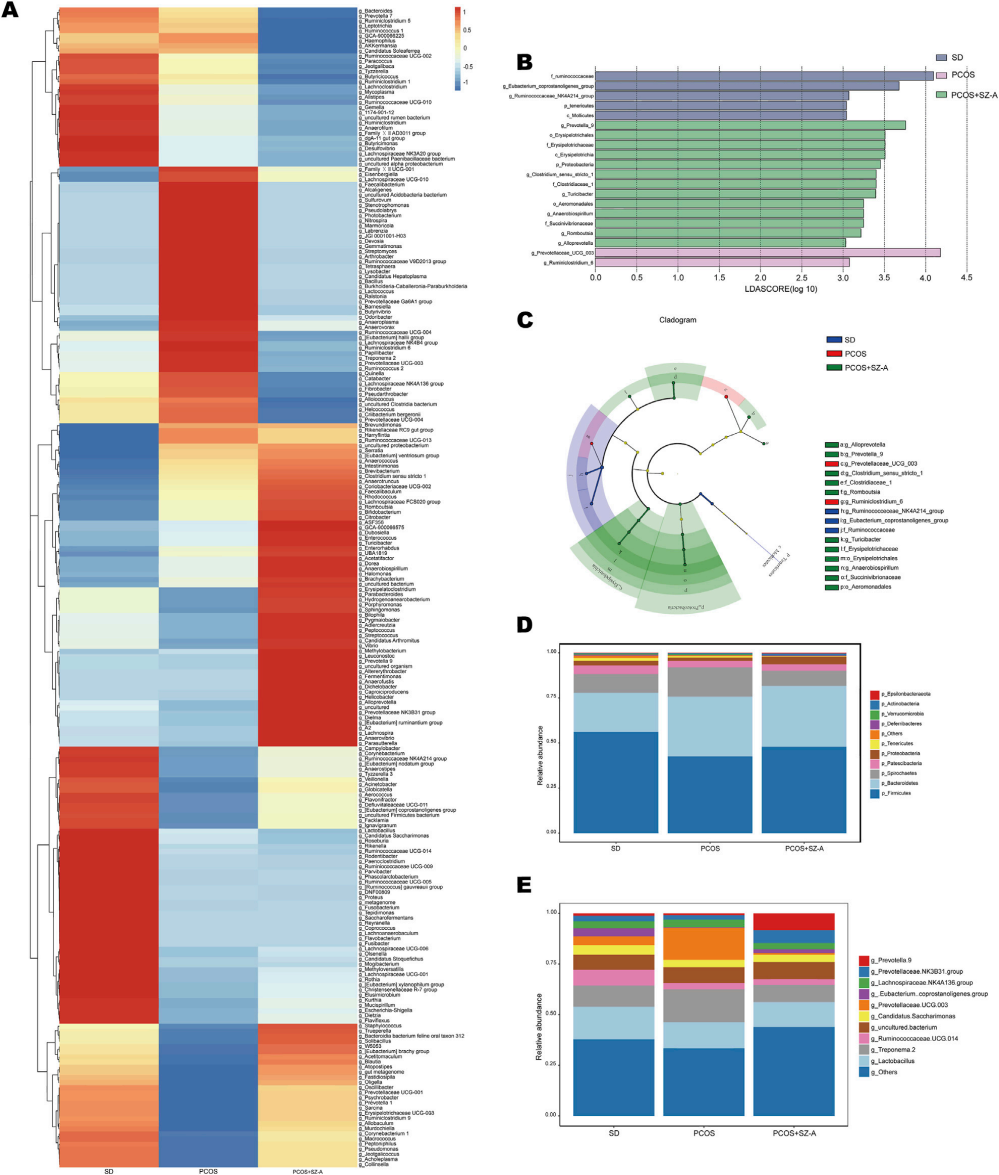
Changes in the taxonomic composition of ileum microbial communities at the phylum and genus levels. **(A)** Heat map at the genus level. **(B)**Statistical differences in the levels of biomarkers between the SD, PCOS, and PCOS+SZ-A groups were identified using line discriminant analysis (LDA) effect size (LEfSe) method. Taxa enriched in SD (Blue), PCOS (purple), and PCOS+SZ-A (Green) groups are indicated by LDA scores. Only the taxa meeting an LDA significant threshold of three are displayed, and the length of histogram represents the influence of different species. **(C)** Cladogram visualizes the output of the LEfSe algorithm. Significantly different classification nodes are colored, and branch regions are colored based on the effect size of the classification group. The top ten bacteria, with maximum abundance of ileum bacteria at the phylum **(D)** and genus **(E)** levels. n = 5 rats in SD group, n = 6 rats in other groups, values are presented as means ± SD.

To determine the primary phylotypes impacted by treatments with DHEA and SZ-A, the confirmed sequences were thoroughly examined using the linear discriminant analysis (Aboeldalyl et al., 2021) effect size (LEfSe) method. The *Eubacterium_coprostanoligenes* and *Ruminococcaceae_NK4A214* genera were enriched in the animals in the SD group, whereas the *Prevotella*, *Clostridium_sensu_stricto*, *Turicibacter*, *Anaerobiospirillum*, *Romboutsia*, and *Alloprevotella* genera were prevalent in the group with PCOS. Additionally, the genera *Prevotellaceae* and *Ruminiclostridium* were predominant in the PCOS+SZ-A group (Figures 5A–C), suggesting that SZ-A could modify the make-up of the intestinal microbiomes of rats with PCOS. Additionally, the composition of the intestinal microbiomes of the three groups was analyzed by determining the taxonomic similarity of the bacteria at the genus and phylum levels. The findings revealed that Bacteroidetes and Firmicutes were the most predominant phyla in all the three experimental groups (Figure 5D), while *Lactobacillus* and *Treponema.2* were the most dominant genera across the three groups at the genus level (Figure 5E).

### 3.5 Relationships between intestinal microbiomes and serum metabolites

**FIGURE 6:**
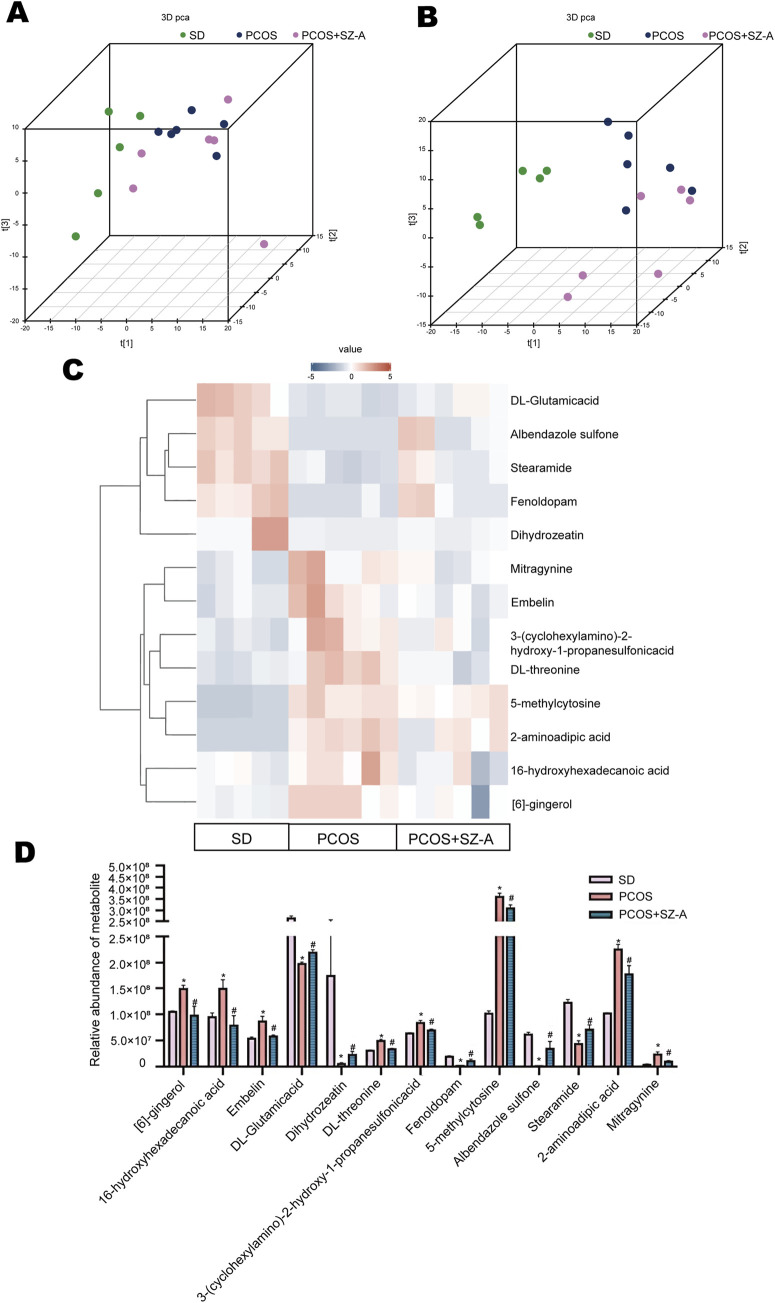
SZ-A affects serum metabolomic profiles. 3D PCoA analysis based on unweighted **(A)** and weighted **(B)** UniFrac distance shows separation of three groups. The color and shape of scatter points represent the experimental grouping of samples. **(C)** An average heat map of the hierarchical clustering analysis for the three groups is presented. Abscissas represent different experimental groups, ordinates represent different metabolites, and different colors represent the relative expression of metabolites at the corresponding position. **(D)** Relative abundance of metabolites between three groups. Values are shown as the mean ± SEM (n = 5 rats in SD group, n = 6 rats in other groups).*represents significance compared to the SD group (*p < 0.05), # represents significance compared to the PCOS group (#p < 0.05). Two-way ANOVA followed by Turkey’s test was used for **(D)**.

The association between the gut microbiomes and the serum metabolites of the three groups was assessed by analyzing the serum metabolites using non-targeted metabolomics studies. Figures 6A,B displays the distinctive differences in primary metabolic components among the groups, as highlighted by principal component analysis (PCA). Notably, SZ-A treatment partially mitigated the alterations in the levels of 13 serum metabolites induced by PCOS. The levels of these 13 metabolites in the three groups are outlined in Figure 6C. For instance, the levels of DL-Glutamic acid, albendazole sulfone, stearamide, fenoldopam, and dihydrozeatin decreased, while [6]-gingerol, 16-hydroxyhexadecanoic acid, embelin, DL-threonine, 3-(cyclohexylamino)-2-hydroxy-1-propanesulfonic acid, 5-methylcytosine, 2-aminoadipic acid, and mitragynine increased in PCOS rats. Following SZ-A treatment, these fluctuations were alleviated (Figure 6D). Our findings suggest that these 13 metabolites could be associated with the positive effects of SZ-A in the PCOS group. Furthermore, DL-Glutamic acid, albendazole sulfone, stearamide, fenoldopam, and dihydrozeatin may contribute positively to PCOS treatment.

**FIGURE 7:**
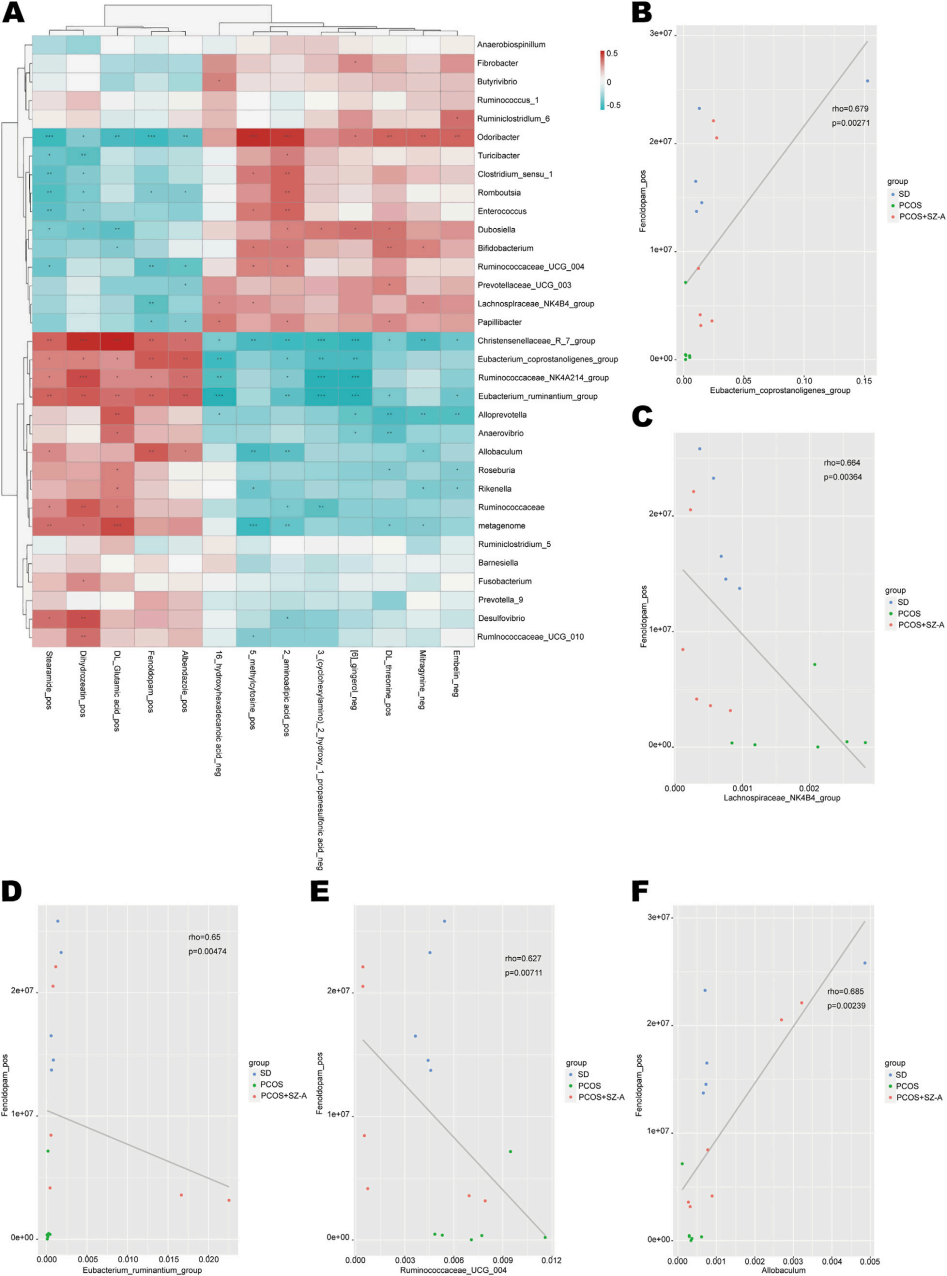
Associations between gut microbial species and circulating metabolites. Spearman’s rank correlation between 33 gut microbial species and 13 metabolites in the SD, PCOS, and PCOS+SZ-A groups is presented **(A)**. Red panes represent positive correlations between bacterial species and metabolites, and blue panes represent negative correlations between bacterial species and metabolites (*p < 0.05; **p < 0.01; ***p < 0.001). Scatter plot indicates the Person’s correlation coefficient with statistical significance (p < 0.05) between Eubacterium_Coprostanogenes **(B)**, Lachnospiraceae_NK4B4 **(C)**, Eubacterium_ruminantium **(D)**, Ruminococcus_UGG_004 **(E)** or Allobaculum **(F)** and serum Fenoldopam levels in all the three groups. n = 5 rats in SD group, n = 6 rats in other groups, values are presented as means ± SD.

Further investigation was conducted on the correlation between the levels of 13 metabolites mentioned earlier and gut microbiota in all three groups through spearman analysis. The findings revealed that 33 bacterial genera were associated with these metabolites (Figure 7A). Among them, Anaerobiospirillum, Ruminococcus_1, Ruminiciostridium_5, Barnesiella, and Prevotella_9 showed no significant correlation with any of the metabolites. On the other hand, Fibrobacter, Butyrivibrio, Ruminococcus_6, Prevotellaceae_UCG_003, *Fusobacterium*, and Ruminococcaceae_UCG_010 displayed significant correlation with one or two metabolites, while Christensenellaceae_R_7_group exhibited significant correlation with all 13 metabolites. Furthermore, the selective dopamine1 (DA1) receptor agonist Fenoldopam was found to be significantly correlated with 10 genera including Eubacterium_Coprostanogenes, Lachnospiraceae_NK4B4, Eubacterium_ruminantium, Ruminococcus_UGG_004, Allobaculum, Christensenellaceae_R_7, Odoriacter, Papillibacter, Romboutsia, and Ruminococcaceae_NK4B4. The correlation between the serum levels of fenoldopam and the 7 significantly correlated genera was visualized with scatter diagrams (**P < 0.01, ***P < 0.001; Figures 7B–F; Supplementary Figures S3A, B). The results demonstrated that low levels of fenoldopam in the sera of rats with PCOS were associated with the reduced abundance of *Eubacterium_Coprostanogenes*, *Eubacterium_ruminantium*, *Allobaculum*, and *Christensenellaceae_R_7*, and the increased abundance of *Odoribacter*, *Ruminococcus_UGG_004*, and *Lachnospiraceae_NK4B4*. Interestingly, intervention with SZ-A led to an increase in serum Fenoldopam levels in PCOS+SZ-A group rats by altering the abundances of these 7 genera.

### 3.6 Fenoldopam mitigates ovarian dysfunction in rats with PCOS

**FIGURE 8:**
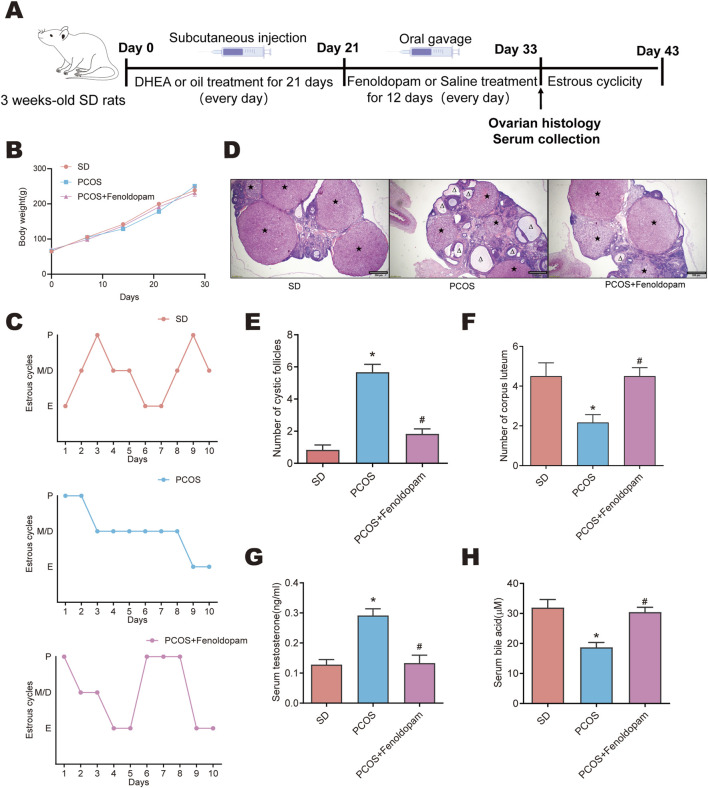
Fenoldopam improved ovarian dysfunction in DHEA-induced PCOS rats. Female rats were randomly divided into three groups: SD, PCOS, PCOS +Fenoldopam. After 21 days of treatment with DHEA or oil, female rats were treated with saline or 100 mg/kg/day Fenoldopam for 12 days. **(A)** Flow chart. **(B)** Body weight. **(C)** The estrous cycles were determined after another 10 days. **(D)** Representative ovarian sections stained with H&E (scale = 200) μ M; △ represents cystic follicles; ★ represents corpus luteum, and determine the number of cystic follicles **(E)** and corpus luteum **(F)** from these stained sections. Serum testosterone **(G)** and bile acid **(H)** levels were measured. Values are shown as the mean ± SEM (n = 6 rats).*represents significance compared to the SD group (*p < 0.05), #represents significance compared to the PCOS group (#p < 0.05). One-way ANOVA followed by Turkey’s test was used for **(E–H)**.

The study aimed to investigate whether Fenoldopam contributes to the positive impacts of SZ-A in rats with PCOS. Rats treated with DHEA received 100 mg/kg/day of Fenoldopam for a duration of 12 days (Figure 8A). The administration of Fenoldopam did not display any noticeable effects on the rats’ body weight (refer to Figure 8B) but did show improvements in estrous cycles irregularities in rats with PCOS (Figure 8C). H&E staining confirmed that the cystic follicular counts in the ovaries decreased following the administration of fenoldopam, while the formation of corpora lutea was elevated after treatment (Figures 8D–F). Additionally, Fenoldopam notably decreased the elevated levels of serum testosterone and restored diminished levels of serum bile acids in rats with PCOS (Figures 8G,H). Altogether, these findings indicate that fenoldopam effectively alleviated ovarian insufficiency and diminished the pathological injury to the ovaries of rats with PCOS.

## 4 Discussion

In the DHEA-induced PCOS rat model, this study systematically evaluated the effects of Ramulus mori (Sangzhi) alkaloids (SZ-A) and demonstrated their potential in restoring reproductive function, alleviating metabolic disturbances, and reshaping gut metabolite interactions. SZ-A treatment not only improved estrous cycle irregularities and ovarian morphology but also reduced androgen levels, elevated circulating bile acids, and markedly mitigated systemic oxidative stress and inflammation. These findings resonate with reports highlighting the gut microbiota-bile acid-IL-22 axis in PCOS (Qi et al., 2019; Geng et al., 2023), suggesting that SZ-A may restore intestinal homeostasis through FXR/TGR5 signaling and downstream immune pathways, thereby indirectly improving ovarian function. In summary, SZ-A represents a promising therapeutic strategy for PCOS with considerable translational potential.

The degree of oxidative stress in patients suffering from PCOS, especially in those with insulin resistance (IR) and obesity-related infertility, is higher than that of healthy individuals, suggesting that oxidative damage is a crucial contributor to the pathophysiology of PCOS (Rudnicka et al., 2022; Zeber-Lubecka et al., 2023). Additionally, the biomarkers of oxidative stress and sex hormones are relevant. The parameters of oxidative stress can serve as biomarkers for the advanced diagnosis of individuals at a high risk of developing PCOS (Yan et al., 2024). Recent research indicates that dysfunctional ovulation might be associated with the onset of oxidative damage in PCOS, and the use of antioxidants can alleviate the status of oxidative stress in individuals with PCOS (Li et al., 2022). A recent study suggested that certain nutrients, including vitamins, minerals, and vitamin analogs, can be administered for the treatment of PCOS (Dubey et al., 2021). These nutritional supplements may ameliorate the consequences of PCOS-induced oxidative injury and aid in alleviating the rates of ovulation and pregnancy in infertile individuals with PCOS (Sandhu et al., 2021).

Interestingly, SZ-A predominantly relieved oxidative stress and inflammation at systemic and intestinal levels, whereas ovarian antioxidant indices remained largely unaffected. This “gut-first” effect implies that the intestine is the primary target of SZ-A (Akbuğa-Schön et al., 2024), with subsequent endocrine and metabolic improvements mediated through microbiota-metabolite interactions. Similar patterns were observed in Tempol-based interventions, where attenuation of intestinal oxidative stress remodeled the microbiota-metabolite interface and ultimately improved PCOS phenotypes (Li et al., 2021). Taken together, these observations support the hypothesis that SZ-A primarily fortifies the intestinal oxidative-inflammatory barrier and subsequently influences systemic and ovarian physiology via bile acid remodeling and microbial metabolites.

Microbiome profiling revealed a significant association between SZ-A treatment and the enrichment of *Christensenellaceae_R-7_group*. This taxon has repeatedly been linked with leanness and metabolic health across human cohorts and animal models, and has been proposed as a directional microbiome signature influencing host metabolism (Akbuğa-Schön et al., 2024; Ignatyeva et al., 2024). Our results showed broad correlations between *Christensenellaceae_R-7_group* and multiple SZ-A-responsive metabolites, suggesting that this lineage may serve as a critical mediator of the metabolic benefits observed. Integrating this bacterial group into the framework of PCOS pathogenesis may help explain SZ-A’s mechanism of action and offers a promising avenue for microbiota-targeted interventions.

Another novel observation emerged from untargeted metabolomics: fenoldopam levels were reduced in PCOS rats but restored following SZ-A treatment, and this metabolite was significantly associated with multiple bacterial taxa. Fenoldopam is a selective dopamine D1 receptor agonist, primarily used clinically for hypertension and renal protection (Cuttone et al., 2024; Esezobor et al., 2024). Importantly, dopamine D1 receptors are expressed in ovarian granulosa cells and are implicated in steroidogenesis and oxidative stress regulation (Alhajeri et al., 2022; Jones-Tabah et al., 2022). Dopamine D1 receptors are Gs coupled and stimulate adenylyl cyclase to raise cAMP, which activates PKA and EPAC, promotes CREB phosphorylation, upregulates steroidogenic machinery such as StAR and CYP11A1, and facilitates mitochondrial cholesterol trafficking to support steroidogenesis (Jones-Tabah et al., 2022; Wang et al., 2024; Shi et al., 2025). In parallel, D1 signaling intersects with ERK and AKT pathways to influence granulosa cell survival and luteinization, while enhancing Nrf2 driven antioxidant responses and, via cAMP and PKA, constraining NLRP3 inflammasome activation, thereby helping preserve ovarian homeostasis (Yang et al., 2021; Bérubé et al., 2024; Zhang L. et al., 2024; Zhao et al., 2025). These findings raise the intriguing possibility that a DA-D1 signaling axis may act as a bridge linking intestinal metabolites to ovarian function. While fenoldopam has not previously been considered in PCOS, its restoration by SZ-A highlights a potential mechanistic pathway warranting validation. Future studies should confirm metabolite identity through authentic standards, followed by pharmacological manipulation with D1 agonists and antagonists in both *in vivo* and *in vitro* systems to test causality.

The modulation of bile acid metabolism by SZ-A also merits attention. Previous work demonstrated that gut colonization with *Bacteroides vulgatus* disrupts bile acid homeostasis and exacerbates PCOS features (Qi et al., 2019), whereas bile acid supplementation or microbial modulation ameliorates ovarian dysfunction (Sun et al., 2023). In our study, SZ-A prevented the decline in bile acid concentrations, suggesting that restoration of the bile acid-FXR/TGR5-IL-22 pathway may underlie improvements in ovarian and systemic outcomes. This interpretation is further supported by reports showing that SZ-A improves bile acid profiles in other metabolic diseases. Thus, SZ-A may act at the intersection of bile acid metabolism, immune signaling, and ovarian physiology (Chen et al., 2022).

Despite these promising insights, several limitations should be acknowledged. First, the DHEA-induced model reflects hyperandrogenic PCOS but does not encompass the full heterogeneity of clinical subtypes. Second, the sample size was relatively small, and microbial analysis was restricted to 16S rRNA sequencing, which limits functional resolution. Third, fenoldopam annotation remains provisional, and direct causal evidence for its role in ovarian function is lacking. Fourth, the causal chain remains incomplete, as causality has not been verified using germ free models or targeted metabolite supplementation add back studies. Future investigations should therefore: (i) employ germ-free or antibiotic-treated models combined with fecal microbiota transplantation to establish microbiota dependence; (ii) use pharmacological interventions in granulosa cells and animal models to define the role of DA-D1 signaling; (iii) integrate transcriptomic and metabolomic profiling to map FXR/TGR5-IL-22 signaling, oxidative stress responses, and microbiota-metabolite networks; (iv) initiate exploratory clinical trials stratified by PCOS subtype, incorporating bile acid profiles, gut microbiota diversity, and metabolite signatures as mechanistic endpoints; and (v) conduct quantitative analysis of primary, secondary, sinusoid, and degenerated follicles to provide a more comprehensive ovarian morphological atlas.

## 5 Conclusion

This study shows that SZ-A provides multifaceted benefits in PCOS by reducing intestinal oxidative stress, reshaping the gut microbiota, restoring bile acid homeostasis, and normalizing metabolite profiles. The identification of Christensenellaceae_R-7_group and fenoldopam as potential mechanistic nodes adds new insight into gut-ovary communication. Rigorous causal studies are needed to validate these findings and to establish SZ-A as a microbiota and metabolism-oriented therapeutic strategy for PCOS.

## Data Availability

The data presented in the study are deposited in the NCBI repository, accession number PRJNA1377144.
