# Correction: Ramulus mori (Sangzhi) alkaloids improve intestinal oxidative damage and inflammation in DHEA-induced polycystic ovary syndrome rats via gut microbiota and metabolite modulation

**DOI:** 10.3389/fphar.2026.1794631

**Published:** 2026-02-04

**Authors:** Yanping Wang, Xianmei Jiang, Shuyi Wu, Qiaohui Wang, Dan Zuo, Biao Huang, Li Jian, Yu Yang, Yong Cai, Xingjian Wen, Ling Yao, Shan Geng

**Affiliations:** 1 Central Laboratory of the People’s Hospital of Dazu, The Affiliated Dazu’s Hospital of Chongqing Medical University, Chongqing, China; 2 Basic Medical College, Chongqing University of Chinese Medicine, Chongqing, China; 3 Clinical Nutrition Department, The Affiliated Dazu’s Hospital of Chongqing Medical University, Chongqing, China; 4 Department of Critical Care Medicine, The Affiliated Dazu’s Hospital of Chongqing Medical University, Chongqing, China; 5 Department of Endocrinology, The Affiliated Dazu’s Hospital of Chongqing Medical University, Chongqing, China; 6 Chongqing Academy of Chinese Materia Medica, Chongqing, China

**Keywords:** Ramulus mori (Sangzhi) alkaloids, PCOS, intestinal oxidative damage, inflammatorystatus, intestinal microbiome and metabolites, fenoldopam, bile acid metabolism

Affiliation 2 “Basic Medical College, Chongqing University of Chinese Medicine, Chongqing, China” was erroneously given as “Basic Medical College, Chongqing College of Traditional Chinese Medicine, Chongqing, China”.

Affiliation 2 “Basic Medical College, Chongqing University of Chinese Medicine, Chongqing, China” was omitted for author Ling Yao. This affiliation has now been added for author Ling Yao.

Author Ling Yao was erroneously assigned to Affiliation 7 “School of Basic Medicine, Chongqing University of Chinese Medicine, Chongqing, China”. This affiliation has now been removed for author Ling Yao.

The original article has been updated.

