# Supplementary material for: Ramulus mori (Sangzhi) alkaloids improve intestinal oxidative damage and inflammation in DHEA-induced polycystic ovary syndrome rats via gut microbiota and metabolite modulation
Source: Front Pharmacol. 2026 Jan 22;16:1701694. doi: 10.3389/fphar.2025.1701694 (PMC12914721; doi:10.3389/fphar.2025.1701694)
Supplement: Supplementary file 1 [file Supplementaryfile1.docx]

Supplementary Material


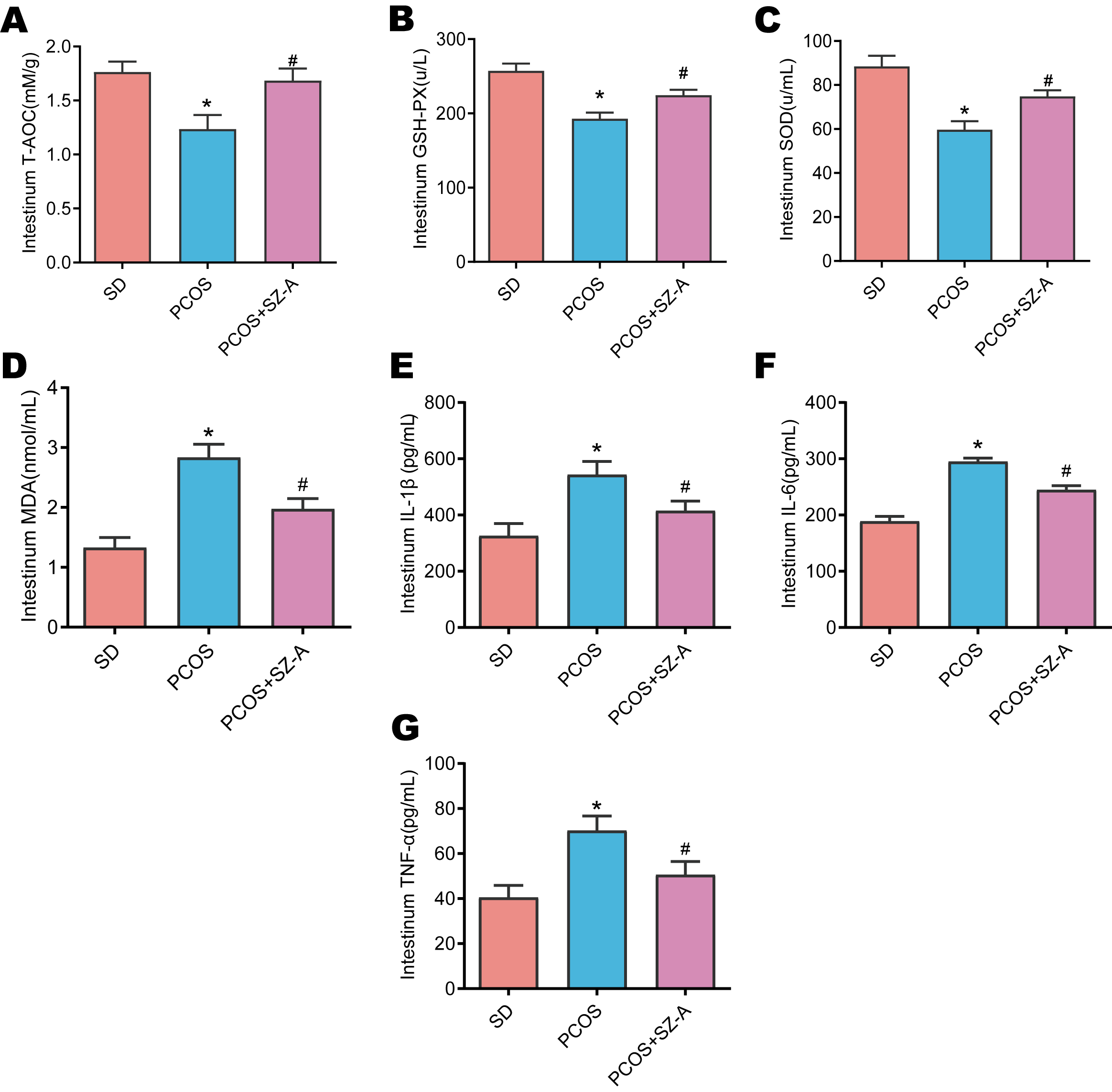


**Figure S1 Effects of SZ-A on intestinal oxidative stress in PCOS rats.** The level of T-AOC (A), GSH-PX (B), MDA(C), SOD activity (D), IL-1 β (E),IL-6 (F) and TNF-α(G) in intestinal tissue lysates were determined. Values are shown as the mean ± SEM (n = 6 rats).*represents significance compared to the SD group (*p<0.05), # represents significance compared to the PCOS group (#p<0.05). One-way ANOVA followed by Turkey’s test was used for all results.


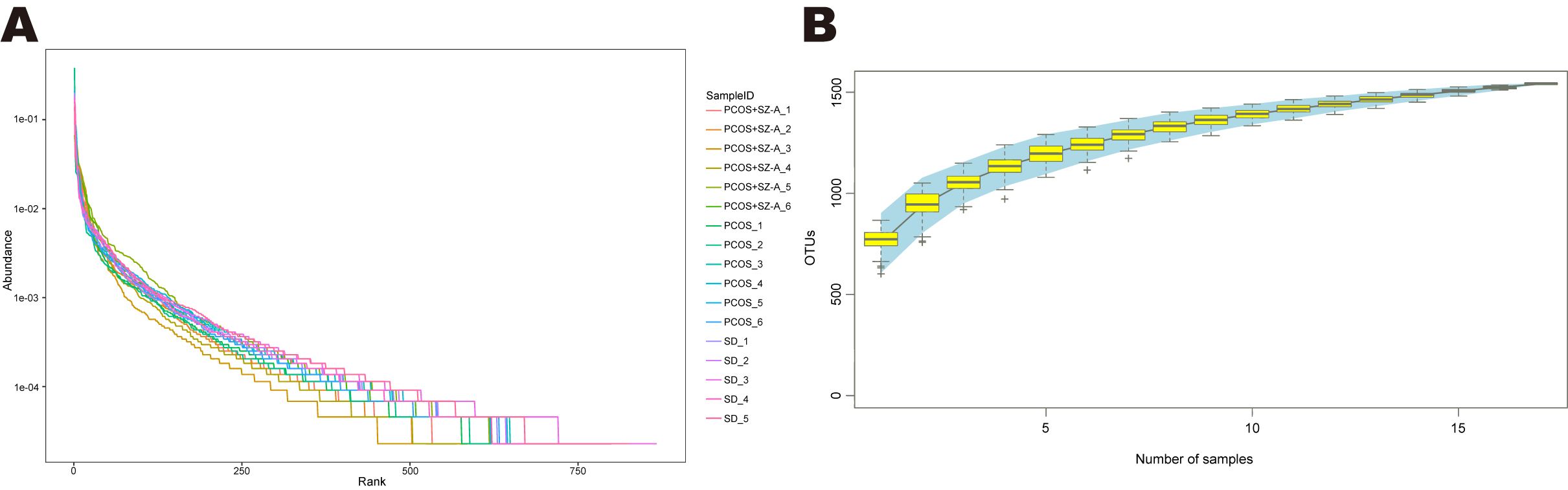


**Figure S2.** (A) Species accumulation. (B) Rank abundance curve..n= 5 rats in SD group, n=6 rats in other groups, values are presented as means ± SD.


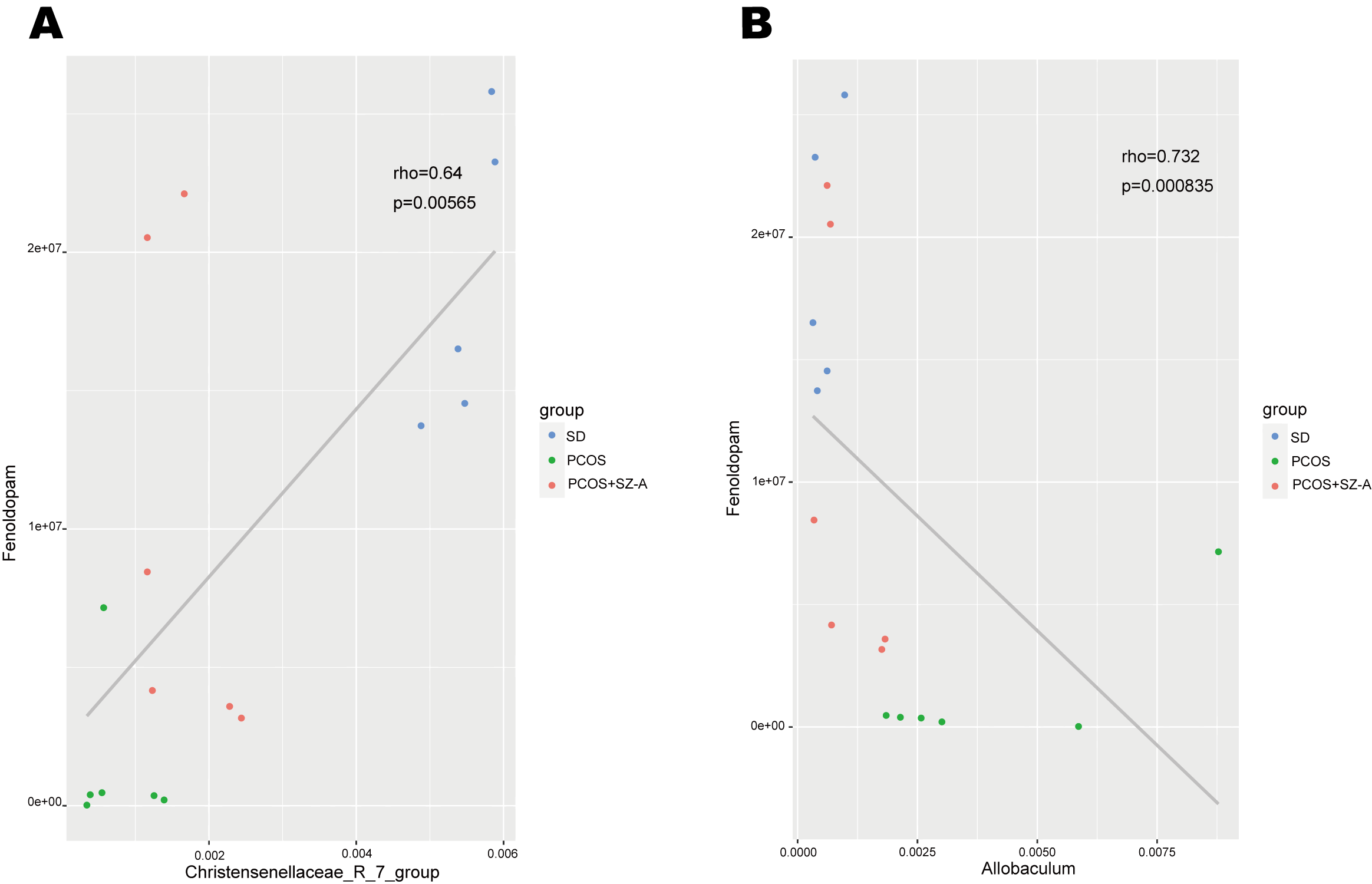


**Figure S3.** Scatter plot indicates the Person’s correlation coefficient with statistical significance (p < 0.05) between Christensenellaceae_R_7(A) or Odoriacter(B) and serum Fenoldopam levels in all the three groups. .n= 5 rats in SD group, n=6 rats in other groups, values are presented as means ± SD.
